# From Inpatient to Ambulatory Care: The Introduction of a Rapid Access Transient Ischaemic Attack Service

**DOI:** 10.3390/healthcare6020057

**Published:** 2018-06-01

**Authors:** Mohana Maddula, Laura Adams, Jonathan Donnelly

**Affiliations:** Tauranga Hospital, Bay of Plenty District Health Board, Tauranga 3112, New Zealand; laura.adams@bopdhb.govt.nz (L.A.); jonathan.donnelly@bopdhb.govt.nz (J.D.)

**Keywords:** transient ischaemic attack, TIA, stroke

## Abstract

*Background*: Transient Ischaemic Attacks (TIA) should be treated as a medical emergency. While high-risk TIAs have higher stroke risks than low-risk patients, there is an inherent limitation to this risk stratification, as some low-risk patients may have undiagnosed high-risk conditions. Inequity of care for TIA patients was observed, such that high-risk patients received urgent assessment through acute admission, while low-risk patients faced long waits for clinical consultation. A redesign of the TIA service was planned to offer timely assessment for all patients and avoid acute admission for high-risk patients. *Methods*: Service reconfiguration was undertaken to set up a daily weekday rapid access TIA clinic where patients would be assessed, investigated, and treated. *Results*: A re-audit of clinic performance showed a significant increase in the number of patients seen in the ages of 18 to 52. The median time from referral to clinical consultation improved from 10 days to 1. There were similar significant improvements seen in median time to brain imaging (from 10.5 days to 1), and carotid ultrasound (from 10 days to all scans being performed on the same day). *Conclusions*: The redesigned service achieved the objective of offering urgent assessment and investigations for all TIA patients, including low-risk patients, while avoiding the acute admission for high-risk patients. We share our experience of establishing a successful rapid access ambulatory service without any additional resources.

## 1. Introduction

Strokes are an important cause of death and long-term adult disability, and many strokes can be prevented with appropriate medical intervention. A Transient Ischaemic Attack (TIA) is defined as an abrupt onset of a focal neurological deficit as a result of focal ischaemia lasting less than 24 h and without radiological evidence of acute ischaemia. TIAs herald high risks of stroke over the next few hours and days, and should therefore be treated as a medical emergency. Urgent clinical assessment, investigation, and initiation of secondary preventive measures have been shown to significantly reduce the risk of stroke [1,2].

A previous New-Zealand-wide audit in 2013 showed evidence of significant improvements in the provision of care for patients with suspected TIA, with a shift to assessment in an ambulatory care setting, rather than acute admission. There was however much variation in practice and service set-up [3]. Management in specialized rapid access TIA clinics results in reduced stroke risk [4]. Moreover, a non-admission-based approach with outpatient clinic follow-up has been shown to be better at stroke prevention and cost-effectiveness, compared with admission-based care [5].

The Tauranga hospital serves a population of about 46,000 in the western Bay of Plenty region of New Zealand. At the time of review, the TIA clinic ran concurrently with the general Stroke & TIA outpatient service, encompassing follow-ups from the inpatient wards and new patient referrals from primary care. ‘High-risk’ TIA patients (those with ABCD2 score > 3, atrial fibrillation or carotid stenosis, crescendo TIAs, and those on anticoagulation) were admitted for urgent investigation and treatment, while ‘low-risk’ TIAs (ABCD2 score < 4) would be mostly managed by GPs, with a follow-up in the hospital TIA clinic.

An audit over two months in 2016 showed unacceptably long delays for review of patients in the TIA clinic and subsequent investigations. There was a concern of underutilization of the service, with the observation that many ‘low-risk’ TIA patients were completely managed by GPs, contrary to current recommendations. There was also an overreliance on the ABCD2 score, with referring clinicians often incorrectly applying this risk-stratification tool to make a TIA diagnosis. The ABCD2 score has low sensitivity and specificity when used by non-specialists in the community or emergency department [6,7]. One in five patients with an ABCD2 score < 4 have symptomatic carotid stenosis of >50% or atrial fibrillation [8]. Over the last few years, there has been a shift away from solely using the ABCD2 score to reliably discriminate between high- and low-risk TIAs. The 2017 Australian & NZ stroke guidelines make a weak recommendation that the ABCD2 score should not be used in isolation when determining the urgency of an assessment, as this may delay the recognition of atrial fibrillation and carotid stenosis [9]. The UK stroke guidelines take one step further and completely abandon use of the ABCD2 score [10].

With this in mind, the TIA service at Tauranga Hospital was redesigned to provide a timely assessment and equity of care for all suspected TIAs, including ‘low-risk’ patients who may have undiagnosed high-risk factors (such as carotid stenosis). Another objective of the new service was to provide rapid ambulatory assessment for ‘high-risk’ patients in place of acute admission.

## 2. Methods

Service reconfiguration was undertaken to set up a daily weekday one-stop rapid access TIA clinic, where patients would be assessed, investigated (on the same day where necessary), and treated. A daily weekday clinic was felt to be needed in order to meet fluctuations in the numbers of referrals. Although desirable to provide a seven days service, this did not prove feasible, as the service was run by only one stroke physician and registrar. Only suspected high-risk TIAs would be admitted during the weekends.

All service improvement projects can face challenges; Table 1 illustrates some of these and how solutions were found. General Practitioner and in-hospital referral pathways were modified, such that high-risk TIA patients would be offered TIA clinic consult within 24–48 h of referral, and low-risk patients within seven days.

## 3. Results

The redesigned TIA service came into effect on 1 May 2017. Referrals were triaged the same way, although the emphasis was put on prompt review of the electronic referral and administrative processes, such as allocation of clinic appointment (Table 1). During the triage, two referrals (both from GPs) were declined following a review of the clinical information, as the likelihood of a neurovascular event or any other serious pathology was felt to be extremely unlikely. In both cases, the referrers were sent prompt written correspondence with advice and signposting of other hospital services if the GP still wanted to a second opinion. Table 2 shows audit results of equivalent time periods before and following the implementation of changes. There was a significant increase in the number of patients seen in the new TIA clinic. Most referrals were from GPs (*n* = 24), followed by ED (Emergency Department) (*n* = 15), other inpatient teams (*n* = 12), and Ophthalmology (*n* = 1). Twenty-four patients and 12 patients were diagnosed with TIA and stroke, respectively; the rest were TIA ‘mimics’, such as syncope, migraine and postural hypotension. Carotid ultrasound imaging was indicated in 25 patients, and in all cases this was performed on the same day. Where indicated, brain imaging was performed on 35 patients: 26 patients had CT (Computed Tomography) scans, and nine had MRIs (Magnetic Resonance Imaging), with a median time of one day. One patient waited 39 days for brain imaging; this was felt to be a non-urgent scan for the evaluation of suspected mild dementia.

**Figure 1:**
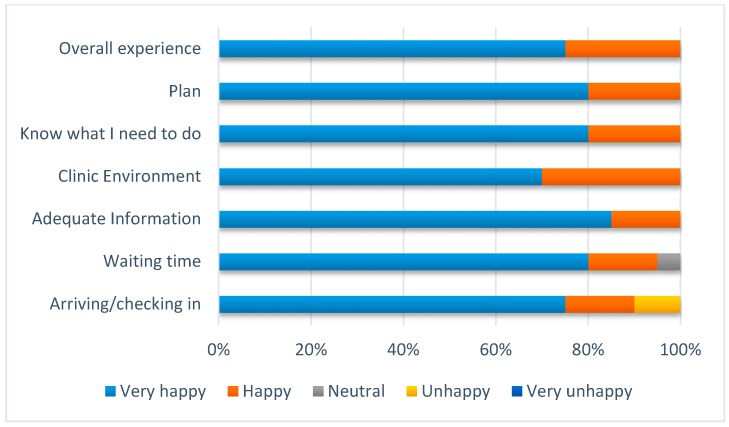
Patient feedback.

There were 21 patients included in the audit classified as high-risk according to the aforementioned criteria, and 31 low-risk patients. For both high- and low-risk patients, the median time to review was one day. No patients were admitted to the hospital with a stroke or TIA within 90 days of their review. Feedback from patients was excellent (Figure 1), despite concern that some may be displeased about having to wait around in the clinic for same-day investigations.

## 4. Discussion

The establishment of rapid access TIA clinic centres across in North America the UK, and Australia has shifted the focus of TIA care from inpatient to outpatient management. In our new rapid access service, all suspected TIA patients were provided with an early consult and underwent timely investigations where needed. We achieved the objective of improving access to specialist assessment for all TIAs, even those deemed to be low-risk, some of which may have yet unknown high-risk conditions. This was achieved while providing timely assessment for high-risk patients without acute admission.

The data shows that there is a substantial reduction in time until specialist consult, time for imaging where required (both brain and carotid), and hospital admissions for high-risk TIAs. Utilisation of the service increased due to an organisation-wide awareness and a shift from community management to rapid specialist referral. A total of 69% of patients seen had a confirmed diagnosis of TIA or stroke, which is in line with similar data obtained from other TIA clinic studies [11]. This would suggest that appropriate patients were being referred to the service, that the triage process worked well to allow prompt clinic review and avoid inappropriate use of the service (two referrals were declined as stated above), and that clinical reasoning and diagnostic accuracy was consistent with standard practice at other centres. One area of concern is the number of patients diagnosed with stroke (12; 23%). While it may be reasonable to provide an outpatient clinic review to those patients who have had a minor stroke, there is a possibility that some patients with acute stroke may not receive the benefit of multidisciplinary team input in a stroke unit if there were seen in clinic. Feedback was provided to GPs and Emergency Department doctors about the importance of referring patients with persisting neurological symptoms for admission, rather than referral to the TIA clinic.

We achieved this service improvement in the provision of timely specialist care for patients with suspected TIA without any additional resources. This was achieved through the redesign of the service, which included the use of electronic systems (such as emails for receipt of referrals instead of hard copy referrals), reducing the inappropriate use of investigations (such as filtering out unnecessary carotid doppler ultrasound requests), and redistribution of existing resource (dropping existing outpatient clinics and using this clinician time to provide the daily TIA service). Although we have not performed a cost-benefit analysis as part of the service redesign, we expect to find cost-saving through the management of suspected high-risk TIA patients through this ambulatory setting instead of hospital admission.

With the ever-increasing burden of healthcare expenditure and demand for hospital beds around the world, healthcare systems are increasingly looking at ambulatory models for the management of various health conditions as an alternative to hospital admission. In the development of our Rapid Access TIA service, we have shared our experience of developing this service together with the challenges we faced and how solutions were found.

## 5. Conclusions

We were able to successfully introduce a rapid access ambulatory clinic for assessment and management of patients with suspected TIA through service redesign. During this process we encountered some challenges, however we were able to find successful solutions without the need for any additional resource.

## Figures and Tables

**Table 1:** Challenges faced by the service improvement project and how solutions were found.

Challenges/Barriers to Change	Solutions
In-hospital processing of referrals was taking a few days as this was reliant on several administrative steps.	The referral process was streamlined; the stroke physician was accessible by phone during working hours and received email alerts of inpatient referrals and electronic General Practitioner referrals, enabling prompt triaging of referrals and early allocation of clinic slots.
Limited outpatient clinic space to see patients at short notice and review after same day investigations.	A single room on the stroke ward was converted into a consultation room. This enabled an easy oversight of clinic patients and a review after investigations.
Limited clinician time. No additional staff were provided.	The Stroke Physician and Registrar dropped one weekly outpatient clinic each and was this time redistributed to provide daily weekday TIA (Transient Ishcaemic Attack) clinics.
Limited Radiology resources. No additional funding to increase carotid or brain imaging appointments.	Referral pathway for carotid imaging was modified, such that only the stroke team could request carotid ultrasound in order to filter out inappropriate investigations (e.g., where imaging was unlikely to change overall management). This allowed for the allocation of two fixed carotid ultrasound appointments per day for TIA clinic patients. Same-day brain imaging was also available.
Need for urgent cardiac monitoring to identify patients with paroxysmal atrial fibrillation/flutter in place of inpatient telemetry.	The Stroke service purchased Holter monitor units to be used for the sole purpose of TIA clinic patients. Patients would have them fitted the same day and wear them for at least 48 h. These would get analysed urgently, and results were forwarded to Stroke physician.

**Table 2:** Audit cycle results before and after implementation of the redesigned service.

	Before 1st Audit Cycle Oct.–Nov. 2016	After 2nd Audit Cycle May–June 2017
Number of patients seen in TIA clinic	18	52
Median time (days) from referral to clinic consult—All referrals	10 (range 0 *–40)	1 (range 0 *–4)
Median time (days) to brain imaging where indicated	10.5 (range 2–64)	1 (range 0 *–39)
Median time (days) to carotid US imaging where indicated	10 (range 1–143)	0 * (all patients imaged on same day)

* same day.

## References

[1] Lavallee P.C., Meseguer E., Abboud H., Cabrejo L., Olivot J.M., Simon O., Mazighi M., Nifle C., Niclot P., Lapergue B. (2007). A transient ischaemic attack clinic with round-the-clock access (SOS-TIA): Feasibility and effects. Lancet Neurol..

[2] Rothwell P.M., Giles M.F., Chandratheva A., Marquardt L., Geraghty O., Redgrave J.N., Lovelock C.E., Binney L.E., Bull L.M., Cuthbertson F.C. (2007). Effect of urgent treatment of transient ischaemic attack and minor stroke on early recurrent stroke (EXPRESS study): A prospective population-based sequential compression. Lancet.

[3] Brownlee W., Ranta A., Dale-Gandar J., Bennett P., Gommans J., Fink J., Barber P.A. (2014). Changes in the provision of transient ischaemic attack services in New Zealand 2008 to 2013. N. Z. Med. J..

[4] Sehatzadeh S. (2015). Is transient ischemic attack a medical emergency? An evidenced-based analysis. Ont. Health Technol. Assess. Ser..

[5] Sanders L.M., Cadilhac D.A., Srikanth V.K., Chong C.P., Phan T.G. (2015). Is nonadmission-based care for TIA patients cost-effective?. Neurol. Clin. Pract..

[6] Bradley D., Cronin S., Kinsella J.A., Tobin W.O., Mahon C., O’Brien M., Lonergan R., Cooney M.T., Kennelly S., Collins D.R. (2013). Frequent inaccuracies in ABCD2 scoring in non-stroke specialists’ referrals to a daily rapid access stroke prevention service. J. Neurol. Sci..

[7] Ghia D., Thomas P., Cordato D., Epstein D., Beran R.G., Cappelen-Smith C., Griffith N., Hanna I., McDougall A., Hodgkinson S.J. (2012). Low positive predictive value of the ABCD2 score in emergency department transient ischaemic attack diagnoses: The south western Sydney transient ischaemic attack study. Intern. Med. J..

[8] Wardlow J.A., Brazzelli M., Chappell F.M., Miranda H., Shuler K., Sandercock P.A., Dennis M.S. (2015). ABCD2 score and secondary stroke prevention. Neurology.

[9] Stroke Foundation (2017). Clinical Guidelines for Stroke Management 2017.

[10] Royal College of Physicians (2016). National Clinical Guideline for Stroke 2016.

[11] Martin P.J., Young G., Enevoldson T.P., Humphrey P.R. (1997). Overdiagnosis of TIA and minor stroke: Experience at a regional neurovascular clinic. QJM.

